# Correction: ‘Drying paint: from micro-scale dynamics to mechanical instabilities’ (2017), by Goehring *et al.*

**DOI:** 10.1098/rsta.2023.0371

**Published:** 2024-01-22

**Authors:** Lucas Goehring, Joaquim Li, Pree-Cha Kiatkirakajorn

**Keywords:** colloids, small-angle X-ray scattering, drying, solidification, fracture, shear bands

*Proc. R. Soc. A.* **375**, 20160161. (Published online 3 April 2017) (https://doi.org/10.1098/rsta.2016.0161)

Due to a calculation error, the experimental data points (red/green circles) reported in figure 8 for *D*/*D*_0_ are incorrect, by a factor of exactly two. The same error is in the accompanying electronic supplementary material, Fig7_8_data_saxs.xlsx, column N. For example, the range of experimental values for figure 8*a* are displayed as *D*/*D*_0_ = 10–25, and this range should be *D*/*D*_0_ = 5–12. The accompanying theoretical calculations (lines in figure) are unchanged by this correction.

